# Diurnal Variation of Serum Total Testosterone in Women: A Single-Center Study From Basrah

**DOI:** 10.7759/cureus.47677

**Published:** 2023-10-25

**Authors:** Hatem D Algburi, Ali Alhamza, Abbas A Mansour

**Affiliations:** 1 Diabetes and Endocrinology, Faiha Specialized Diabetes, Endocrine and Metabolism Center, Basrah, IRQ; 2 Diabetes and Endocrinology, University of Basrah, College of Medicine, Basrah, IRQ

**Keywords:** non-fasting, women, basrah, testosterone, diurnal variation

## Abstract

Background

The diurnal variation of testosterone in women has received limited attention, despite its growing recognition as a crucial factor in female health and well-being. This study aims to investigate the diurnal fluctuations of total testosterone levels in apparently healthy women with regular menstrual cycles.

Methodology

A cross-sectional study was conducted at Faiha Specialized Diabetes Endocrine and Metabolism Center in July 2023. This study involved 46 apparently healthy women volunteers aged between 21 and 40 years. To explore diurnal variations in total testosterone, blood samples were collected from each participant at two distinct time points, i.e., 8:30 AM and 1:30 PM. These samples were collected regardless of fasting status with the exclusion of the menstruating phase.

Results

The mean total testosterone level at 8:30 AM was 23.4 ± 12.4 ng/dL and at 1:30 PM was 21.7 ± 12.9 ng/dL, with a p-value of 0.03. Neither age nor body mass index demonstrated a significant impact on testosterone levels.

Conclusions

This study showed a significant diurnal variation in serum total testosterone levels among apparently healthy women, with higher levels observed in the morning.

## Introduction

Testosterone, a key hormone primarily associated with male physiology, has long been recognized for its role in various aspects of human health and behavior. While testosterone is predominantly produced in the testes of males, it is also synthesized in smaller amounts by the ovaries, adrenal glands, and peripheral conversion in females. Traditionally, testosterone has been considered a male hormone; however, recent research has shed light on its importance and influence on women’s health and well-being [1-3]. Although women produce much lower levels of testosterone compared to men, it is still a significant hormone in their bodies. Testosterone in women has various physiological processes and influences overall well-being. Studies have indicated that testosterone is involved in maintaining bone density, muscle mass, and cognitive function in women [2,4]. Additionally, it plays a role in sexual desire and arousal, as well as mood regulation. Moreover, testosterone levels can vary across the menstrual cycle, with fluctuations at different phases impacting women’s mood and behavior [5]. Understanding the role of testosterone in women is essential for comprehending the intricacies of female hormonal health and its implications for overall health and quality of life [2]. Historically, the role of testosterone in women has been overshadowed by its prominent effects in males, leading to limited research focused on understanding its dynamics within the female body. However, recent advances in endocrinology and the growing recognition of the multifaceted functions of testosterone have sparked interest in exploring its significance in women’s health [2]. The circadian rhythm, or the diurnal variation of hormones, plays a pivotal role in maintaining the body’s homeostasis and orchestrating various physiological functions across the 24-hour day-night cycle [6]. The diurnal variation, or daily fluctuation, of testosterone levels has been extensively studied in men and is well-documented. Testosterone levels in men exhibit a characteristic pattern, with peak concentrations occurring in the morning and declining gradually throughout the day [7]. This diurnal rhythm of testosterone has been linked to various physiological processes, including energy metabolism, sexual function, and mood regulation. However, the diurnal variation of testosterone in women remains relatively understudied, despite its potential implications for female health and reproductive function [7]. In men, testosterone has been extensively studied in the context of diurnal variation, with testosterone levels known to exhibit a characteristic pattern, peaking in the early morning and gradually declining throughout the day [7,8]. Nevertheless, the diurnal variation of testosterone in women has been relatively understudied, despite the emerging understanding of its importance in female health and well-being [9,10]. Understanding the diurnal variation of testosterone in women is essential, as it may provide valuable insights into the hormonal dynamics underlying various physiological and psychological processes. Hormonal fluctuations in women have been extensively investigated in the context of menstrual cycles, with estrogen and progesterone being the primary focus. However, the diurnal variation of testosterone in women remains less explored [9]. Limited research suggests that women may also exhibit a diurnal rhythm in testosterone levels similar to men. However, the magnitude and pattern of this variation, as well as the factors influencing it, are not well understood. Unraveling the diurnal variation of testosterone in women could have significant implications for a range of health-related areas, such as reproductive function, bone density, muscle mass, cognitive performance, and overall well-being [10,11].

The objective of this study is to investigate the diurnal variation of total testosterone in apparently healthy regularly cycling women.

## Materials and methods

A cross-sectional study was undertaken at Faiha Specialized Diabetes, Endocrine and Metabolism Center (FDEMC) in July 2023. The study comprised 46 apparently healthy female volunteers from the medical personnel of FDEMC, aged between 21 and 40 years, all of whom provided written consent to participate in the research.

We included healthy normal menstruating women with no previous diseases aged 21-40 years with regular cycles (21-35 days) regardless of the menstrual phase and working time shift. The following patients were excluded: women aged less than 21 years or more than 40 years, women in their menstrual periods, women with polycystic ovary disease, women with hereditary adrenal disease (e.g., congenital adrenal hyperplasia), those taking medicines that affect serum testosterone such as contraceptive pills, and those with systemic diseases that affect serum testosterone levels (e.g., chronic kidney disease, chronic liver disease, inflammatory bowel disease, and acute illness).

We conducted a clinical evaluation of women, excluding cases that did not meet the specified research conditions. Additionally, we performed anthropometric measurements for weight and height to calculate body mass index (BMI). To investigate the diurnal variation of total testosterone, blood samples were drawn from each participant at two different time points, i.e., 8:30 in the morning and 1:30 in the afternoon. The women did not need to be in a fasting state and could be in the follicular or luteal phase but not in the menstruating phase. These specific time points were chosen to capture any fluctuations that might occur during the day. Serum total testosterone levels were assessed using electro-chemiluminescence immuno-assay kits on the Cobas e411 analyzer series (Roche Diagnostics, Basel, Switzerland). The assay was designed with a defined range of 2.5-1,500 ng/dL, featuring an intra-assay precision within the range of 15-50 ng/dL and exhibiting a coefficient of variation (CV) of ≤10%. The normal range of total testosterone in women was determined according to Braunstein et al. [12]. The estimated 5th and 95th percentiles for a 30-year-old woman were 15-46 ng/dL [12].

Statistical analysis

SPSS version 26 (IBM Corp., Armonk, NY, USA) was used to determine the standard deviation and the mean for normally distributed numerical data and the median and percentage for categorical data. The data were normally distributed. Accordingly, a paired-sample t-test was applied to measure the differences in the means of the two groups. P-values less than 0.05 were considered significant.

## Results

In total, 46 women were enrolled with a mean age of 28.4 ± 4.7 years. Overall, 67.4% were single, with a mean BMI of 26.1 ± 4.2 kg/m^2^ (Table 1). The mean testosterone at 8:30 AM was 23.4 ± 12.4 ng/dL and at 1:30 pm was 21.7 ± 12.9 ng/dL.

**Table 1:** General characteristics of participants.

Variables	
Age (years), mean ± SD	28.4 ± 4.7
Age range (years)	21–40
Unmarried (%)	31 (67.4)
Married (%)	15 (32.6)
Height (cm), mean ± SD	156.3 ± 16.9
Weight (kg), mean ± SD	68.0 ± 16.9
Body mass index, mean ± SD (kg/m^2^)	26.1 ± 4.2
Body mass index, range (kg/m^2^)	16.02–38.40
Testosterone 8:30 AM (ng/dL), mean ± SD	23.4 ± 12.4
Testosterone 1:30 PM (ng/dL), mean ± SD	21.7 ± 12.9
Total number (%)	46 (100)

On a paired-sample t-test, the mean morning testosterone level was significantly higher than the mean afternoon testosterone level (mean difference = 1.77 ± 5.38 ng/dL, p = 0.03). The inter-individual CV of testosterone among the participants was 53.1% and 59.7% in the morning and afternoon, respectively. The intra-individual CV for the same person at different times was 12%, as shown in Table 2.

**Table 2:** Comparison of means AM testosterone and PM testosterone (ng/dL). Data are expressed as mean ± standard error of the mean. Δ testosterone: the difference between serum total testosterone at 8:30 AM and 1:30 PM; BMI: body mass index; CV: coefficient of variation

Variable	Mean	SD	SE	Inter-individual CV%	Intra-individual CV%
Testosterone 8:30 AM (ng/dL)	23.47	12.46	1.83	53.1%	12%
Testosterone 1:30 PM (ng/dL)	21.70	12.97	1.913	59.7%	
Δ Testosterone AM and PM (ng/dL)	1.77	5.38	0.79	-	
P-value	0.03			0.351	

However, we did not observe any significant effects of age or BMI on the diurnal variation of total testosterone, as seen in Table 3.

**Table 3:** Effect of age and BMI on Δ testosterone (ng/dL). Data are expressed as mean ± standard error of the mean. Δ testosterone: the difference between serum total testosterone at 8:30 AM and 1:30 PM; BMI: body mass index

Parameters	Age groups	Δ Testosterone (ng/dL)	P-value
Age (years)	≥28	2.6 ± 0.7	0.282
	<28	0.8 ± 1.5	
BMI (kg/m^2)^	≥25	1.2 ± 0.8	0.381
	<25	2.7 ± 1.6	

## Discussion

Diurnal variation of testosterone is well described in men but not in women [9]. In women, testosterone measurement is affected by food, phase of the menstrual cycle, and BMI with a weak level of evidence [13]. Measurement of testosterone at a low level is far from perfect, especially when using immunoassay because of limited method accuracy, precision, sensitivity, and specificity to measure hormones at a low level [14,15]. We found some diurnal variation of total testosterone in this study in apparently healthy women with a modest drop in total testosterone at PM. A similar finding was also seen in women with polycystic ovary disease [10]. In this study, testosterone measurements were conducted without considering the fasting state. This approach was chosen due to the lack of clear data regarding testosterone measurement in women while fasting, in contrast to the well-established guidelines for men [16]. A previous study conducted by Ali et al. at the same center focused on apparently healthy women aged 18-45 years. It reported that serum total testosterone levels were elevated in the fasting state for healthy women but did not observe the same effect in women with hirsutism or menstrual irregularities [17]. We measured total testosterone in this study regardless of the menstrual cycle phase because of the small study sample, despite the suggestion that total testosterone varies in women according to menstrual cycle phases [18]. Age was not associated with an increase in testosterone in this study. A previous study found that androgen decreases with age. This difference was likely due to the study sample or the narrow age range in our study (21-40 years) [19]. The increasing level of total testosterone with increased BMI was seen before in hirsute women but not seen in our study because of the study sample or enrolment of healthy women only [20]. The lack of a significant relationship between testosterone levels and BMI highlights the complexity of hormonal regulation in women’s bodies. Further research is warranted to explore the underlying factors that govern testosterone fluctuations and their impact on women’s health. Seasonal variation of testosterone is also seen in men, with the highest in August-October declining after and the lowest in March. This study was conducted in July 2023. Further studies taking into consideration the season need to be conducted in the future.

Limitations

This study assessed testosterone levels within a limited timeframe of five hours, primarily due to logistical constraints. The measurements were not conducted during the fasting state or the menstrual cycle due to the small sample size. Testosterone was quantified using an immunoassay method, as the liquid chromatography and tandem mass spectrometry assay were unavailable; however, the immunoassay was considered an acceptable alternative [21].

## Conclusions

The study proved that there is a difference between testosterone levels in the morning compared to the evening from a statistical standpoint. Studies are needed with a larger number of relatively healthy participants, with assessment at a perfect time, i.e., 4 PM or later, and dividing them into two phases of the menstrual cycle through which the clinical significance can be proved.
